# Supplementary material for: Role of Aryl Hydrocarbon Receptor Activation and Autophagy in Psoriasis-Related Inflammation
Source: Int J Mol Sci. 2020 Mar 22;21(6):2195. doi: 10.3390/ijms21062195 (PMC7139675; doi:10.3390/ijms21062195)
Supplement: Supplementary file 1 [file ijms-21-02195-s001.pdf]

International Journal of *Molecular Sciences*

Supplementary Materials

Table S1. The effects of M5 on chemokines and antimicrobial peptide production [16].

	Keratinocyte cultures	Human skin explants	Animal model
CXCL chemokine production	CXCL1, CXCL5, CXCL8 ↑	CXCL8 ↑	CXCL1, CXCL2, CXCL3 ↑
Chemotactic activity	Neutrophil chemotactic activity↑		
Antimicrobial peptide production and activity	BD2, BD3, S100A7 ↑<br>Antibacterial activity to *E.coli* ↑	BD2, S100A7 ↑	BD2, S100A7 ↑
Transcriptional profile change	BD2, BD3, LL37, RNASE7, PI3, S100A7, S100A7A, S100A12, CXCL1, CXCL2, CXCL3, CXCL5, CXCL6, CXCL8 ↑		

Table S2. mRNA primers.

Gene	Product number
AHR	TaqMan Assay ID Hs00169233_m1
CYP1A1	TaqMan Assay ID Hs1054794_m1
LC3	TaqMan Assay ID Hs00176567_m1
ATG5	TaqMan Assay ID Hs00169468_m1
BECN1	TaqMan Assay ID Hs01007018_m1
IL-1β	TaqMan Assay ID Hs1555410_m1
IL-6	TaqMan Assay ID Hs00174131_m1
TNF-α	TaqMan Assay ID Hs00174128_m1
GAPDH	TaqMan Assay ID 02758991_m1

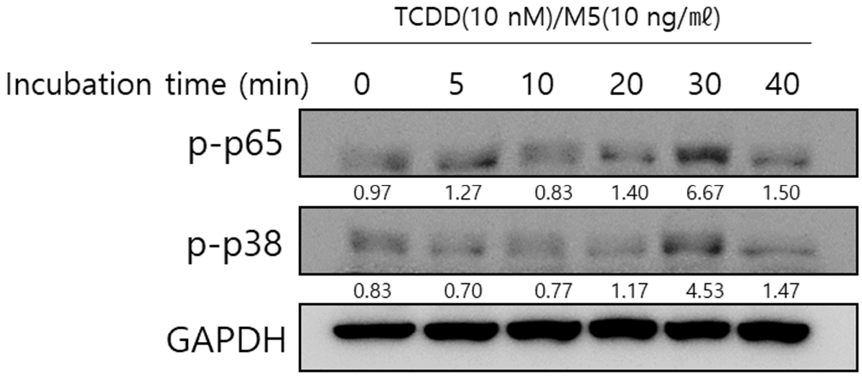

Figure S1. In M5-stimulated HaCaT cells, TCDD induced p65NF-κB and p38MAPK phosphorylation; the effect peaked at 30 min. HaCaT cells were exposed to serum-free medium containing TCDD (10 nM) and M5 (10 ng/mL) for different time periods (0, 5, 10, 20, 30, and 40 min). Cell lysates from each sample were used for western blot analyses with antibodies directed against p-p65NF-κB and p38MAPK.The density of the phosphorylated p38MAPK and p65NF-κB bands was normalized to the corresponding loading controls. The results are representative of three independent experiments.
